# Editorial processing of *Epidemiologia e Serviços de Saúde:* advances and perspectives in 2025

**DOI:** 10.1590/S2237-96222025v34e20252002.en

**Published:** 2025-08-11

**Authors:** Jorge Otávio Maia Barreto, Everton Nunes da Silva, Maria Auxiliadora Parreiras Martins, Wildo Navegantes de Araújo, Taís Freire Galvão

**Affiliations:** 1 Fundação Oswaldo Cruz, Gerência Regional de Brasília, Brasília, DF, Brazil; 2 Universidade de Brasília, Faculdade de Ciências e Tecnologias em Saúde, Brasília, DF, Brazil; 3 Universidade Federal de Minas Gerais, Faculdade de Farmácia, Belo Horizonte, MG, Brazil; 4 Universidade Estadual de Campinas, Faculdade de Ciências Farmacêuticas, Campinas, SP, Brazil

In June 2025, we completed our first year as editors of *Epidemiologia e Serviços de Saúde: revista do SUS* (RESS)-a period of intense activity during which we sought to incorporate best editorial practices to improve processing, based on recommendations from the scientific community and scientific evidence ^1^. When we took on this commitment, we were fully aware of the professional challenge it posed, as well as of the importance of honoring the legacy of this journal, which has over three decades of history and is one of the leading publications in the field of public health in Brazil. On the eve of this first year of editorial activities, it seems appropriate to reflect on the results obtained and the prospects outlined for the journal.

In 2024, RESS published 104 scientific manuscripts, including original articles, systematic reviews, and research notes, as well as three peer-reviewed opinions. All of them are available in full on the journal’s SciELO portal. Of these, 30 articles comprised the special issue *20 Years of Trans Visibility in Brazil* , and 25 comprised the special issue *Vaccination Coverage Survey* *for Children Born in 2017 and 2018*. In the first half of 2025, RESS had already published 52 articles and 68 peer-reviewed reports, all included in vol. 34. Aside from maintaining quantitative indicators that exceed the requirements of the SciELO Brazil Collection ^2^, our editorial practices primarily aimed to improve the experience of authors, reviewers, and editors ^3^-an outcome reflected in processing time, which decreased from an average of 172 days in 2023 to 85 in 2024, and less than 60 in the first half of 2025. The analysis and feedback on quantitative indicators enable the editorial team to monitor and address potential bottlenecks in the editorial workflow; however, this process can-and should-also be enriched by the experience of authors and reviewers. Comments and suggestions are always welcome and may be sent directly to the journal’s Editorial Office or the editors through the corresponding author’s contact.

In the second half of 2025, we will begin publishing editorial reports written by RESS editors, further enhancing transparency and alignment with open science practices. Making reviewer and editor reports publicly available tends to increase their engagement and the quality of their evaluations. Together with information on data availability-which has been required since the second half of 2024, following SciELO Criteria ^2^-this environment fosters good communication practices. It enhances the scientific integrity of research published in RESS. 

An open call for new editors was issued to increase diversity and provide researchers from across the country the opportunity to join the editorial team ^4^. These editors will receive training on the peer review process. The aim is to expand the candidates’ skills and ensure equitable access for all applicants, regardless of prior experience, region, or institutional affiliation. Encouraging the use of a standardized peer review form also helps structure the reports and improve communication with authors, who are more likely to receive comprehensive and constructive feedback for manuscript improvement ^3^.

Additionally, two special calls for papers are currently underway as part of strategic partnerships with the Brazilian National Health System (*Sistema Único de Saúde*, SUS) and the Ministry of Health. The first call welcomed manuscripts on evidence-informed policy studies in Brazil, supported by the Ministry of Health through the Evidence-Informed Policy Network (EVIPNet), coordinated in Brazil by the Ministry itself ^5^. The second special call in 2025 will accept manuscripts reporting field research from the Field Epidemiology Training Program for SUS Health Services (*Programa de Treinamento em Epidemiologia Aplicada aos Serviços do SUS*, EpiSUS), in celebration of its 25th anniversary ^6^. Both calls highlight scientific output that is applied to local realities and that enhances decision-making within the SUS, fostering the interface between epidemiology and health services. 

Other initiatives are underway to further disseminate information that supports evidence-informed decision-making in the SUS, such as the creation of the “Guideline” modality in RESS, which will allow for the publication of recommendations on health practices within the SUS. These may include documents that will be part of the 7th edition of the *Guia de Vigilância em Saúde* (*Health Surveillance Guide*), a strategic publication of the Ministry of Health’s Secretariat of Health and Environmental Surveillance ^7^.

These activities strengthen the scope of RESS and its role as the SUS journal, which “publishes scientific articles in the field of public health, including epidemiology, social and human sciences in health, management, and planning, addressing topics relevant to the SUS.” The definition of RESS’s scope in 2024 was the result of a broad discussion during the update of the instructions for authors. It filled an information gap, meeting a specific criterion of the SciELO Brazil Collection ^2^. The definition of the scope and its implementation in the activities developed and planned align with the *Fifth Master Plan for the Development of Epidemiology in Brazil 2025-2029*, prepared by the Epidemiology Committee of the Brazilian Association of Collective Health (*Associação Brasileira de Saúde Coletiva*, Abrasco) ^8^.

We invite the scientific community and SUS professionals to submit research contributions that reflect the pillars of this plan in the areas of academic training, research, and epidemiology, particularly concerning health policies, programs, and services.

## References

[1] Galvão TF, Silva END, Araújo WN, Barreto JOM (2024). Editorial improvement in Epidemiologia e Serviços de Saúde in 2024. Epidemiol Serv Saude.

[2] SciELO (2024). Critérios, política e procedimentos para a admissão e a permanência de periódicos na Coleção SciELO Brasil.

[3] Silva MT, Galvao TF (2024). Systematization of peer review in Epidemiologia e Serviços de Saúde. Epidemiol Serv Saude.

[4] Epidemiologia e Serviços de Saúde: revista do SUS (2025). Chamada pública para editores associados da RESS.

[5] de Oliveira S, Bento AL, Valdes G, de Oliveira STP, de Souza AS, Barreto JOM (2020). Institutionalizing evidence-based policies in Brazil. Rev Panam Salud Publica.

[6] Brasil, Ministério da Saúde, Secretaria de Vigilância em Saúde (2025). EpiSUS.

[7] Brasil, Ministério da Saúde, Secretaria de Vigilância em Saúde e Ambiente (2024). Guia de Vigilância em Saúde.

[8] Araújo T, Ramos A, Boing A, Moraes C, Melo E, Nery J (2025). V Plano Diretor para o Desenvolvimento da Epidemiologia no Brasil - 2025-2029.

