# Supplementary material for: Editorial processing of Epidemiologia e Serviços de Saúde: advances and perspectives in 2025
Source: Epidemiol Serv Saude. 2025 Aug 11;34:e20252002. doi: 10.1590/S2237-96222025v34e20252002.en (PMC12342846; doi:10.1590/S2237-96222025v34e20252002.en)
Supplement: Translation

# Translation: Processamento editorial da Epidemiologia e Serviços de Saúde: avanços e perspectivas em 2025

**DOI:** 10.1590/S2237-96222025v34e20252002.pt

**Authors:** Jorge Otávio Maia Barreto, Everton Nunes da Silva, Maria Auxiliadora Parreiras Martins, Wildo Navegantes de Araújo, Taís Freire Galvão

**Affiliations:** 1 Fundação Oswaldo Cruz, Gerência Regional de Brasília, Brasília, DF, Brasil; 2 Universidade de Brasília, Faculdade de Ciências e Tecnologias em Saúde, Brasília, DF, Brasil; 3 Universidade Federal de Minas Gerais, Faculdade de Farmácia, Belo Horizonte, MG, Brasil; 4 Universidade Estadual de Campinas, Faculdade de Ciências Farmacêuticas, Campinas, SP, Brasil

Em junho de 2025, completamos um ano à frente da *Epidemiologia e Serviços de Saúde: revista do SUS* (RESS), período de intensas atividades, em que buscamos incorporar as melhores práticas editoriais para aperfeiçoar o processamento, com base em recomendações da comunidade e em evidências científicas ^1^. Ao assumirmos esse compromisso, tivemos clareza do desafio profissional que se colocava, bem como da importância de honrarmos o legado desse periódico com mais de três décadas de história, e que figura entre os principais da saúde coletiva do Brasil. Na ocasião desse primeiro ano de atividades editoriais, é oportuno refletir sobre os resultados obtidos e acerca das perspectivas traçadas para a revista.

Em 2024, a RESS publicou 104 artigos científicos, incluindo artigos originais, revisões sistemáticas e notas de pesquisa, além de três pareceres de revisão por pares, disponíveis na íntegra no portal SciELO da revista. Desse total, 30 artigos integraram o número especial *20 anos da visibilidade trans no Brasil* , e 25, o *número especial Inquérito de cobertura vacinal* *de crianças nascidas em 2017 e 2018*. No primeiro semestre de 2025, a RESS já publicou 52 artigos e 68 pareceres de revisão por pares, disponíveis no vol. 34. Além de manter indicadores quantitativos que superam os requisitos da Coleção SciELO Brasil ^2^, as práticas editoriais visaram principalmente aprimorar a experiência de autores, revisores e editores ^3^, o que se evidenciou no tempo de processamento, que passou de 172 dias em média, em 2023, para 85 em 2024, e menos de 60 no primeiro semestre de 2025. A análise e retroalimentação de indicadores quantitativos permite ao corpo editorial observar e corrigir eventuais gargalos no fluxo editorial, mas também é um processo que pode e deve ser enriquecido pela experiência dos autores e revisores, com comentários e sugestões, que são sempre bem-vindos e podem ser encaminhados diretamente à Secretaria da revista, ou aos editores, por meio de contato com o autor correspondente.

No segundo semestre de 2025, iniciaremos a publicação de pareceres de editores da RESS, o que trará ainda mais transparência e alinhamento às práticas de ciência aberta. Disponibilizar ao público os pareceres de revisores e editores tende a aumentar o comprometimento desses profissionais e a qualidade de suas avaliações. Em conjunto com a informação de disponibilidade dos dados do artigo - exigida desde o segundo semestre de 2024 e em atendimento aos Critérios SciELO ^2^ -, esse ambiente favorece boas práticas de comunicação e aumenta a integridade científica das pesquisas veiculadas na RESS. 

Uma chamada aberta para novos editores foi publicada, com vistas a aumentar a diversidade e dar a pesquisadores de todo o país a oportunidade de compor o corpo editorial ^4^. Esses editores receberão treinamento sobre o processo de revisão por pares; com isso, busca-se ampliar as competências dos candidatos, garantindo acesso equitativo entre os inscritos no certame, independentemente de experiência, região e instituição de filiação. O estímulo ao uso de ferramenta padronizada para elaboração dos pareceres também favorece a estruturação dos pareceres e a comunicação com os autores, que potencialmente receberão comentários completos e construtivos para aprimoramento dos manuscritos após revisão ^3^.

Ademais, estão em andamento duas chamadas especiais de artigos que representam parcerias estratégicas para o Sistema Único de Saúde (SUS) e o Ministério da Saúde. A primeira acolheu manuscritos sobre estudos no campo das políticas informadas por evidências no Brasil fomentadas pelo Ministério da Saúde, no âmbito da Rede de Políticas Informadas por Evidências (EVIPNet), coordenada no Brasil por esse ministério ^5^. A segunda chamada especial de 2025 receberá manuscritos de pesquisas de campo do Programa de Treinamento em Epidemiologia Aplicada aos Serviços do SUS (EpiSUS), em comemoração aos seus 25 anos ^6^. Ambas as chamadas valorizam a produção científica aplicada à realidade dos territórios e o aprimoramento das decisões no SUS, promovendo a interface entre a epidemiologia e os serviços de saúde. 

Outras iniciativas seguem em andamento, no intuito de aumentar a disseminação de informações que apoiem as decisões informadas por evidências no SUS, como a criação da modalidade “Diretriz” na RESS, voltada a disseminar condutas em saúde no âmbito do SUS, abrindo a possibilidade de publicação de orientações que comporão a 7ª edição do *Guia de Vigilância em Saúde*, publicação estratégica da Secretaria de Vigilância em Saúde e Ambiente do Ministério da Saúde ^7^.

Tais atividades fortalecem o escopo da RESS e seu papel de revista do SUS, que “publica artigos científicos no campo da saúde coletiva, incluindo epidemiologia, ciências sociais e humanas na saúde, gestão e planejamento, e que abordem temáticas relevantes para o SUS”. A definição do escopo da RESS, em 2024, se deu após ampla discussão para atualização das instruções aos autores e supriu uma lacuna de informação, atendendo a um critério específico da Coleção SciELO Brasil ^2^. A definição do escopo e sua materialização nas atividades desenvolvidas e planejadas estão em consonância com o *V Plano Diretor para o Desenvolvimento da Epidemiologia no Brasil 2025/2029*, elaborado pela Comissão de Epidemiologia da Associação Brasileira de Saúde Coletiva (Abrasco) ^8^.

Convidamos a comunidade científica e os profissionais do SUS a apresentar contribuições de pesquisa que reverberem os eixos desse plano nas áreas temáticas de formação, pesquisa e epidemiologia em políticas, programas e serviços de saúde.
